# Community nurses’ experiences of receiving targeted support in a regional nursing consortium: a qualitative study

**DOI:** 10.3389/fpubh.2025.1664569

**Published:** 2025-10-06

**Authors:** Wen lin Cheng, Shuo Wang, DongMei Zhang, Rui Li, Kaixin Bian, Jia Zhang, Yifan Zhang, Jin Tu, Meijun Yan, Jun Zhou, Shuang Yuan Song

**Affiliations:** ^1^Department of Nursing, Tongren Hospital Shanghai Jiao Tong University School of Medicine, Shanghai, China; ^2^Department of Nursing, Beihua University, Jilin, China; ^3^Community Health Service Center, Shanghai, China; ^4^Department of Nursing, Huayang Street Community Health Service Center, Shanghai, China

**Keywords:** community nursing, regional nursing consortium, mentoring model, qualitative study, healthcare workforce

## Abstract

**Background:**

Regional nursing consortia aim to optimize nursing resource allocation and enhance the quality of primary care. However, community nurses often encounter challenges that diminish their motivation and professional satisfaction, hindering effective implementation. Notably, their lived experiences remain under-explored.

**Aim:**

To explore community nurses’ experiences of receiving support within regional nursing consortia, to improve the model’s effectiveness and sustainability.

**Methods:**

Between November and December 2024, 162 community nurses affiliated with a regional nursing consortium were considered eligible. From this pool, 10 nurses were purposively selected for in-depth interviews until data saturation was reached. The interviews were analyzed using Colaizzi’s phenomenological method.

**Results:**

Three major themes and ten subthemes emerged: (1) Nurses acknowledged the mentoring model’s positive effects, including expanded practice scope, professional development, and increased confidence in managing complex cases. (2) Enthusiasm for active participation was low due to heavy workloads, stress, and dissatisfaction with performance-based pay. (3) Nurses held high expectations for the model, including broader project coverage, varied support formats, insurance-funded services, clearer role boundaries, and expanded patient engagement.

**Conclusion:**

While community nurses value the support provided through the regional nursing consortium, their engagement remains limited. To enhance participation, initiatives should reduce workload, diversify support approaches, and expand project scope. Strengthening professional development and improving incentive mechanisms are essential. Additionally, outreach efforts targeting patients and families should be increased to maximize the model’s impact.

## Introduction

As the “Action Plan for Further Improving Nursing Services (2023–2025)” jointly issued by the National Health Commission and other ministries is being implemented in depth, the formation of medical consortia has become a pivotal direction in the current healthcare system reform. This initiative aims to channel high-quality nursing resources from large-scale hospitals downwards, bolstering the nursing service capabilities of both medical institutions and grassroots units within the region (1). Nursing consortia, a crucial subset of medical consortia, focus on optimizing nursing resource allocation. Their goals are to enhance the quality and efficiency of grassroots nursing services, satisfy the increasingly diverse and multi-level health demands of the public, and resolve the “last-mile” issue of professional nursing services (2, 3). In this context, the regional-based nursing consortium model has been developed (4). This model forges strong partnerships between tertiary-level hospitals and grassroots hospitals or nursing homes, creating nursing consortia to enable the sharing and optimal allocation of nursing resources. Despite notable achievements in some regions through active exploration and practice, the development of nursing consortia in China is still in its nascent stage, with room for substantial improvement in implementation levels (5). During the actual operation, community nurses encounter numerous challenges in the process of receiving support and guidance. These issues not only dampen the work enthusiasm and professional fulfilment of community nurses but also impede the effective implementation of the nursing consortium model and the continuous enhancement of grassroots nursing services. Employing qualitative research methods, this study endeavors to gain an in-depth understanding of the support-receiving experiences of community nurses within the regional-based nursing consortium model. By doing so, it aims to unearth the primary problems, challenges, and successful experiences encountered by community nurses during practice. The findings of this study are expected to offer both theoretical underpinnings and practical references for further optimizing the nursing consortium model, refining its framework, ensuring its stable operation, and ultimately elevating the quality of grassroots nursing services.

In addition to policy-level initiatives, empirical data highlight the urgency of addressing workforce challenges in community nursing. National surveys report that China faces a shortage of qualified nurses, with a nurse-to-population ratio of 3.7 per 1,000 in 2022—lower than the OECD average of 8.8 per 1,000 (2). Community health service centers, in particular, remain understaffed, with some reporting nurse vacancy rates above 20% (5). Excessive workloads have also been documented: community nurses often manage over 50 patient visits per week, in addition to administrative and outreach tasks (1). Such demands contribute to high levels of occupational stress and burnout; one multicenter survey found that more than 60% of Chinese community nurses reported moderate-to-high work-related stress, and 45% reported symptoms of emotional exhaustion (6). These statistics underscore the systemic pressures shaping community nurses’ experiences and provide essential context for interpreting the present study’s findings.

To provide more clarity, the regional-based nursing consortium in this study was established to strengthen the professional capacity of community nurses through structured training and mentorship. The consortium was designed and led by a tertiary hospital, with objectives to: (1) expand the scope of community nursing practice, (2) enhance chronic disease and wound care management capabilities, (3) improve referral efficiency and continuity of care, and (4) foster professional confidence through expert-guided mentoring. Support was delivered through multiple formats, including regular on-site ward rounds by hospital specialists, themed workshops, online interactive lectures, case discussions, and simulation-based training. Community nurses were expected to participate actively in these activities, integrate newly acquired knowledge into their daily practice, and provide feedback for continuous improvement of the program. Each participant was assigned to a senior mentor, responsible for guiding clinical decision-making, evaluating practical skills, and promoting professional growth.

## Materials and methods

### Study participants

From November to December 2024, 162 community nurses certified by our hospital and participating in the regional nursing consortium were considered eligible. Using purposive sampling, 10 participants were ultimately recruited to ensure diversity in age, years of service, job position, community unit, duration of being mentored, and number of times being mentored. Recruitment continued until thematic saturation was achieved, meaning that no new codes or themes emerged from the interviews.

Many frontline and early-career nurses declined due to workload constraints, and male nurses were not available in comparable positions within the participating consortium at the time of recruitment. Therefore, the final sample consisted of females community nurses. While this sample size represents a small proportion of eligible nurses, recruitment continued until thematic saturation was reached, ensuring that no new themes emerged from additional data. This approach is consistent with qualitative research standards prioritizing depth of insight over numerical representation.

The inclusion criteria were as follows: (1) Community nurses who were certified by our hospital and participated in the community nursing union model of the regional base; (2) Having been mentored for more than 3 months under the nursing union model; (3) Having no adverse records during the mentoring process; (4) Consenting to participate in this interview.

Within the consortium, participants were required to attend monthly training sessions, take part in at least one case-sharing or consultation activity each quarter, and engage in supervised clinical practice sessions. They were also expected to document their learning outcomes and demonstrate the application of acquired skills in community settings, which were periodically evaluated by their mentors.

### Development of the interview outline

The research team first developed an interview outline according to research goals and a review of relevant literature. After discussing with senior community nursing experts, the outline was refined. After preliminary interviews with two community nurses, the outline structure was adjusted and finalized. The final one includes: Do you know what a nursing union is? Share your understanding. What problems did you encounter during support receiving? What help did you get from the nursing union? What suggestions do you have for its future development?

### Data collection and quality control

The interview outline was constructed based on the research objectives and determined through expert consultation. The interviewers were experienced and rigorously trained researchers who established a trusting relationship with the interviewees. The same researcher was responsible for both the interview record-taking and the transcription. The transcription was completed immediately after the interview by combining the audio recordings with the recollection of the interview scenarios. Communicative validity verification was adopted, and the interviewees’ recognition of the content of the transcribed materials was obtained through a second meeting. After the data were transcribed, they were re-verified, and reflective records were used to enhance the reliability of the entire study.

### Statistical analysis

Immediately after the interviews, transcription was carried out to prevent information loss. During transcription, the audio recordings were listened to repeatedly, and combined with the interview notes and a review of the interview scenarios, the interview recordings were transformed into written materials. After transcription, data were analyzed using Colaizzi’s seven-step phenomenological methods (6, 7). First, two researchers (WL.C. and R.L.) independently read all transcripts multiple times to gain a full understanding. Second, significant statements relevant to the study objectives were extracted. Third, these statements were coded into meaningful units. Each coder conducted this process independently, and coding reliability was ensured through cross-checking. Fourth, recurring viewpoints were clustered into categories, which were then summarized into subthemes. Fifth, detailed and comprehensive descriptions were written to capture the essence of each category. Sixth, the subthemes were integrated into broader themes. Coding discrepancies were discussed among the two coders, and a third senior researcher (S.W.) was consulted to reach consensus when disagreements occurred. Seventh, results were returned to participants for validation through follow-up meetings, ensuring credibility and accuracy. Throughout the process, reflective notes and an audit trail were maintained to enhance dependability and confirmability of the findings.

### Ethical considerations

Participants were informed of the purpose, significance, and methods of the study. Confidentiality was ensured by using coded identifiers, and informed consent was obtained prior to conducting and recording the interviews. This study has been approved by the Ethics Committee of Beihua University (approval no. 447-FG/BU).

## Results

Ten participants were included in this study, and their names were replaced with identifiers N1-N10. All ten participants were female, with ages ranging from 40 to 51 years old and years of service ranging from 17 to 25 years. All ten participants were directors of community nursing departments. Among them, three were chief nurses, three were associate chief nurses, and four were charge nurses. The duration of being mentored was 6 months for all. The maximum number of times being mentored was 10, the minimum was 6, and the average number of times being mentored was 8. The relevant information about the participants is shown in Table 1. The interview data were extracted and summarized thematically (Table 2).

**Table 1:** Sociodemographic and clinical characteristics of participants.

Encodings	(a person’s) age (years)	Length of service (years)	Title	Duties	Community unit	Duration of mentoring (months)	Number of mentoring sessions attended
N1	41	23	Head physio-therapists	Director of Nursing	Huayang Street Community Health Service Center	6 months	10
N2	45	19	Head physio-therapists	Director of Nursing	Zhoujiaqiao Street Community Health Service Center	6 months	8
N3	51	30	Deputy director physio-therapists	Director of Nursing	Chengjiaqiao Street Community Health Service Center	6 months	8
N4	42	25	Deputy director physio-therapists	Director of Nursing	Hongqiao Street Community Health Service Center	6 months	7
N5	44	17	Head Physio-therapists	Director of Nursing	Beixinjing Street Community Health Service Center	6 months	7
N6	45	24	Supervisors Physio-therapists	Director of Nursing	Tianshan Road Street Community Health Service Center	6 months	7
N7	44	21	Deputy director physio-therapists	Director of Nursing	Xianxia Street Community Health Service Center	6 months	8
N8	44	22	Supervisors physio-therapists	Director of Nursing	Xinjing Town Community Health Service Center	6 months	9
N9	47	23	Supervisors physiotherapists	Director of Nursing	Xinhua Street Community Health Service Center	6 months	10
N10	40	20	Supervisors physiotherapists	Director of Nursing	Jiangsu Street Community Health Service Center	6 months	6

**Table 2:** Themes and sub-themes of the study.

Theme	Sub-theme
Community nurses’ recognition of the positive effects of the mentoring model under the regional base nursing union	Expansion of the scope of practice, like a “cocoon—breaking” process
	Enhanced development of professional knowledge and skills
	Improved confidence in managing complex nursing challenges
Community nurses’ low willingness to actively participate in the mentoring model of the regional base nursing union	Cumulative increase in workload and pressure
	Noticeable reduction in performance-based pay compared to pay.
Community nurses have high hopes for the mentoring model under the regional base nursing union	Desire for broader mentoring projects coverage
	Call for diversified mentoring approaches
	Expectation of medical insurance coverage for nursing services
	Need for clear role boundaries and professional authority
	Expansion of patient education and outreach strategies

### Theme 1: Community nurses’ recognition of the positive effects of the mentoring model under the regional-base nursing union

#### Subtheme 1: Expansion of professional practice scope

Three interviewees indicated that under the mentoring of the regional base nursing union model, the boundaries and scope of their practice had been expanded. N2: “Compared with previous community work, since the implementation of the regional base nursing union model, I have had the opportunity to participate in specialized outpatient services”. N7: “The ‘nursing union’ model has enabled me to participate in specialized projects of wound and stoma care, no longer being confined to basic nursing”. N9: “Previously, routine nursing operations were the majority. Now, with the help of this model, I have joined chronic disease management. I can also collaborate with superior hospital institutions through the Internet and participate in referral tracking and other work”.

#### Subtheme 2: “Rapid growth” development of professional knowledge and skills

Half of the interviewees reported that under the mentoring of the regional base nursing union model, their professional knowledge and skills had significantly improved. Interviewee 01 said, “The nursing union model allowed me to join an online special course on diabetes nursing and learn first-aid skills. Now, I can adjust care plans for diabetic patients and manage emergencies more confidently”. N4: “Last week, a community resident had an accidental injury that caused a long-term non-healing leg wound. But I had previously participated in specialized wound care training and learned the use of new-type wound dressings and infection-prevention nursing techniques”. N6: “Under the leadership of the nursing union model, the average score of our community nurses in professional knowledge assessments has increased from 71 points previously to 85 points now. This progress is truly remarkable”.

#### Sub-theme 3: Enhanced confidence in handling difficult nursing problems

Under the mentoring of the regional base nursing union model, community nurses have become more confident in handling difficult nursing problems. N3: “Previously, whenever I encountered slightly complex problems, I would feel nervous. But now, through expert consultations and joint nursing ward rounds, for example, when dealing with severe pressure ulcer nursing problems, I applied the methods learned from the platform, and the rehabilitation effect was quite good. Now, I’m no longer afraid; instead, I feel very capable”. N4: “In the past, when facing thorny nursing problems, I always had a sense of ‘being left alone.’ Now, I know that there is a ‘nursing union’ behind us, and a group of professional partners are supporting me”. N8: “I can clearly feel that our community nurses are more confident. When I made rounds in various specialized outpatient clinics, I saw our nurses making full use of the knowledge they learned from the nursing union to help patients solve problems. The patients’ positive feedback has increased their confidence and made them more convinced that the future development of community nursing is promising”.

### Theme 2: Community nurses’ low willingness to actively participate in the mentoring model of the regional base nursing union

#### Subtheme 1: Cumulative increase in workload and pressure

Under the mentoring model of the regional base nursing union, community nurses reported an increase in workload and work pressure. N1: “Since the union model began, administrative tasks increased significantly, often leaving me no time for meals”. N2: “The community is indeed developing towards high-quality development, but the human resources in the community are limited. In addition to maintaining the normal operation of daily outpatient clinics, wards, and specialized outpatient clinics, we also have to participate in nursing work such as family sickbeds. Moreover, the ‘Internet + nursing service’ is a home-visit nursing service, and there is a severe shortage of staff”. N3: “Under this assistance model, the daily working hours have been extended, and the rest time has been compressed. Sometimes, we even need to participate in various training courses and meetings on rest days. I feel that my mental state is highly tense, and this will dampen everyone’s enthusiasm for actively participating in the assistance model”.

#### Subtheme 2: Noticeable reduction in performance-based pay

N2: “It is obvious that the workload is much more than before, and I am more tired, but the department’s performance-based salary received each month has not increased. I think my efforts are not proportional to my income, and I feel that the performance-based pay distribution is unreasonable”. N7: “I originally thought that the assistance model could bring new development opportunities, but the performance-based pay distribution is in a mess. For example, our promotion mechanism is not linked to the results of the assistance, and I feel that my future is very uncertain”. N8: “During the assistance process, I was in charge of the management and tracking of chronic diseases, and the results were remarkable, but the performance-based bonus points were pitifully few. Instead, some simple administrative affairs are given more bonus points. I hope that, in addition to the ‘nursing union’ assistance project, a more reasonable salary management model can be provided”.

### Theme 3: Community nurses have high hopes for the mentoring model under the regional base nursing union

#### Subtheme 1: Desire for broader mentoring projects coverage

Some community nurses indicated that the content of mentoring projects needs to be further expanded to meet the broader care needs of the community. N2: “Currently, the main skills that the ‘nursing union’ mentors us in the community are PICC maintenance and wound and stoma maintenance. However, I think there are many patients with multiple co-existing diseases in the community. Risk assessment methods and measures for various aspects such as falls, nutrition, thrombosis, and infections could also be included in the mentoring for us”. N6: “With the help of the regional base nursing union, I have indeed made great progress. But as the seasons change, seasonal diseases such as sudden allergies occur frequently in the community, and my ability to deal with these is still very weak. I eagerly hope that this content can be added to the mentoring projects”. N7: “There are many disabled older adult people in our community now. For example, I have not fully mastered the key knowledge of how to guide them to carry out effective limb nursing rehabilitation training to maintain muscle strength”.

#### Subtheme 2: Call for diversified mentoring approaches

Three community nurses said that the mentoring forms and methods need to be further innovated. Interviewee 01 said, “Currently, the forms of mentoring in our community are relatively fixed. More case-sharing meetings could be organized for us to share experiences. I also hope to have one to one mentor guidance to solve individual practical operation problems and enable us to grow faster”. Interviewee 06 stated, “Regional mentoring is a good thing, but currently, there are mostly theoretical lectures. I’m looking forward to an increase in simulated scenario training, such as emergency treatment simulations for sudden diseases, and the launch of group competition—based learning to stimulate everyone’s enthusiasm”. Interviewee 08 mentioned, “It would be great if there could be an online platform for real—time interactive questions answers, so that we can immediately consult nursing experts from superior hospitals when we encounter problems at work”.

#### Subtheme 3: Expectation of medical insurance coverage for nursing services

All community nurses expressed their hope and appeal that the assistance projects be included in medical insurance. Interviewee 01 said, “Under the regional base nursing union model, we can provide a lot of professional nursing services for residents, such as wound dressing changes. If these can be included in medical insurance, the burden on residents will be reduced, and more people will come to the community for nursing care”. Interviewee 03 stated, “While serving in the community every day, I find that the nursing costs for many older adult people with chronic diseases are quite high. If these long-term nursing projects can be covered by medical insurance, their economic pressure will be much reduced, and our work will be more meaningful”. Interviewee 09 mentioned, “Including the mentoring projects in medical insurance, I believe this is the common expectation of everyone and also an urgent issue”.

#### Subtheme 4: Need for clear role boundaries and professional authority

Some community nurses expressed their hope to clarify the scope of practice authority and services, and establish positive and negative lists. Interviewee 06 said, “We need policy support. There should be a clear boundary regarding what we can and cannot do. Within the scope of what we can do, we will provide services to the best of our ability. Having precise and clear practice authority can also avoid some risks and potential hazards”. Interviewee 04 stated, “When we conduct regular follow-up visits for chronic disease patients, we want to ask more questions, but we are afraid of overstepping the boundaries. It would be great if there were regulations specifying what we can and cannot do”. Interviewee 02 mentioned, “The issue of prescription rights has always troubled community nurses. We hope that at the national policy level, we can have certain authority to use drugs within our capabilities”.

#### Subtheme 5: Expansion of patient education and outreach strategies

Some community nurses expressed their hope to strengthen publicity among patients and their families. Interviewee 07 said, “Patients should also know about the ‘nursing union’, so they can trust our training feel confident seeking care”. Interviewee 09 stated, “In reality, the awareness of patients and their families about some of the new service contents we carry out is relatively low. For example, they do not understand the role of establishing health management files and the significance of regular follow-up visits. We should increase publicity to let them understand our intentions and professionalism”. Interviewee 10 mentioned, “The nursing services of the ‘nursing union’ can be promoted and publicized through online subscription-based account push and community activities to improve the public’s awareness and acceptance of this service model, and let them understand the advantages and convenience of this service”.

## Discussion

Community nurses recognize the positive effects of the mentoring model under the regional base nursing union, but their actual participation is low. Community nurses recognize the positive effects of the mentoring model under the regional base nursing union. It can expand the boundaries and scope of their practice, enhance professional knowledge and skills, and boost their confidence in handling difficult nursing problems. The possible reasons for this are as follows: With the transformation of the disease spectrum and health management model, after chronic disease patients pass the acute-phase in the hospital, their subsequent rehabilitation and nursing are mainly completed in the community and at home. Therefore, the transfer from the hospital to the community and family is the focus of current nursing research in China (8). Meanwhile, under the background of “Healthy China,” a series of health policies vigorously advocate promoting the improvement of community care capabilities through the downward flow of high-quality nursing resources from hospitals (9, 10). The implementation of the “hospital-community” linkage project has effectively improved the nursing theoretical knowledge and common chronic disease nursing skills of community nurses to a certain extent. The regional linkage training has effectively enhanced the chronic disease nursing skills, rehabilitation nursing skills, and emergency nursing skills of community nurses, and the regional linkage project has been recognized by community nurses.

Although community nurses recognize the positive effects of the mentoring model under the regional base nursing union, their actual willingness to participate actively is not high. This interview result is similar to the research by Zhang Xiaochen. Among 196 nurses from 14 community health service institutions in Minhang District, Shanghai, they recognized the “Internet + nursing service” activities carried out by the nursing union, but the actual participation willingness rate was only 54.08% (11). The reasons for the low willingness of community nurses to participate in the mentoring model of the nursing union may be related to factors such as gender differences, uneven human resource allocation, and performance-based pay distribution. All the research subjects in this survey are female, and their willingness to participate actively is low. However, a survey found that male nurses have a higher willingness rate to participate in the “Internet + nursing service” carried out by the nursing union, possibly because the proportion of male nurses among community nurses is low, resulting in less competition pressure (12). Besides gender differences, uneven human resource allocation and performance-based pay distribution are also important factors affecting the willingness of community nurses to actively participate in the nursing union. Under the call for the high-quality development of community health, the nursing manpower in the community is gradually becoming tight. Community nurses need to maintain the normal operation of daily outpatient clinics, wards, and medical service stations, and also participate in nursing work such as family doctor studios and family sickbeds. In addition, participating in the work of the nursing union requires more time, which increases the challenge of community nursing human resource allocation (11) and reduces the willingness of community nurses to actively participate in the nursing union projects. The development of effective nursing services is inseparable from a scientific assessment mechanism (13). However, currently, some assessments deviate from the actual work, such as emphasizing assessment rather than implementation, having excessive frequencies, overly empty indicators, overly complicated processes, and overly high standards, which greatly affect the work willingness and enthusiasm of nurses (14). Therefore, optimizing the allocation of community nurse human resources reasonably and improving the scientific performance—based pay distribution can enhance the enthusiasm of community nurses to actively participate in nursing union projects and encourage them to provide higher—quality nursing services (15, 16).

Beyond the descriptive accounts of increased workload and reduced motivation, these findings can also be understood within broader systemic and organizational contexts. The low enthusiasm of community nurses reflects structural challenges such as inadequate human resource allocation, insufficient staffing to match growing service demands, and performance-based pay mechanisms that fail to reward additional responsibilities (3). From the perspective of Herzberg’s motivation-hygiene theory, inadequate compensation and heavy workload represent “hygiene factors” that, if not addressed, reduce job satisfaction and undermine motivation. Similarly, the Job Demands–Resources model suggests that excessive demands (e.g., administrative burden, extended hours) without corresponding resources (e.g., staff support, fair incentives) lead to strain and disengagement. This interpretation highlights that the reluctance of community nurses to actively participate in the consortium is not simply an individual attitude problem, but a reflection of organizational misalignment and systemic constraints (12). Addressing these factors requires structural reforms in workforce planning, compensation systems, and supportive policies, rather than solely relying on individual motivation or training initiatives.

Although several barriers were identified, our analysis suggests that excessive workload and limited human resources should be prioritized as the most critical issue (7). Nearly all participants emphasized the strain caused by expanded responsibilities without corresponding staffing support, which in turn amplified stress and made dissatisfaction with pay more pronounced. This indicates that workload functions as a root cause, while stress and perceived unfairness in performance-based pay are secondary effects that exacerbate disengagement (2). From a policy perspective, interventions should therefore begin with optimizing workforce allocation and reducing administrative burden, as these changes could directly alleviate stress and improve perceptions of fairness in compensation (10). Once staffing adequacy is addressed, refining incentive mechanisms and creating structured psychological support systems can further strengthen motivation and engagement. Prioritizing workload reduction thus represents the most immediate and impactful strategy for improving participation in the consortium.

There are deficiencies in the actual operation of the assistance model under the regional base nursing union, which need to be improved. The nursing union in China started relatively late, and there are many deficiencies in its development process. Some community nurses reported in the interviews that the assistance forms under the nursing union model are relatively limited, which cannot mobilize the initiative of community nurses, and there are many blocking factors in the actual participation in regional linkage projects. At present, the flexibility of the mentoring model of the nursing union in China is insufficient. It mainly relies on centralized short-term training and special lectures. The time of such training is limited, and more practical training methods such as nursing ward rounds, nursing consultations, on-site guidance, and scenario simulations are less applied. At the same time, the opportunities for community nurses to participate in regional nursing union projects are unequal, the training process lacks full—process management, the retraining mechanism is lacking, and there is no irregular assessment mechanism, resulting in a significant reduction in the training effect and making it difficult to be effectively implemented.

However, some research has provided new ideas for the development of the nursing union. Gui and Liu (17) believe that the training duration has no significant impact on the core competencies of community nurses. Excessive training time may instead cause work pressure and learning burnout among nurses, and online self—learning lacks an effective supervision and feedback system. After combing the education and training systems for community nurses abroad, Sun et al. (18) proposed that training programs should be formulated according to the individual differences of nurses. Mathieson (19) constructed a standardized training system integrating theoretical, experimental, and practical teaching; Zhou et al. (20) continuously improved the traditional Chinese medicine nursing service capabilities of community nurses through the “hospital—community” linkage mode, covering basic training, clinical practice training, intensive training, follow—up feedback, and retraining; Wang et al. (21) used a software system to create a regional homogeneous linkage mode, and adopted diversified training forms such as micro—lecture—based flipped classrooms, case databases, and workshops, which effectively improved the wound nursing skills and clinical nursing capabilities of community nurses and promoted the homogeneous development of the knowledge and skills of community nurses. Therefore, the relevant management personnel of the nursing union should attach great importance to the effect feedback and retraining of the nursing ability training of community nurses under the mentoring model of the nursing union, enhance the flexibility of the linkage forms, smooth the communication channels, and effectively implement the promotion of the linkage projects to improve the level of community nursing services.

This study identified three themes: recognition of the mentoring model’s benefits, limited willingness to participate, and high expectations for future improvements. Excessive workload and inadequate staffing emerged as the most critical barriers, with dissatisfaction over pay and limited patient engagement as secondary challenges. Policy implications include optimizing workforce allocation, reforming incentive systems, expanding project scope with flexible formats, and integrating services into medical insurance. Although implementation costs were not directly assessed, prior evidence suggests investments in mentoring and training may be offset by reduced hospital readmissions and improved chronic disease management. Future evaluations should incorporate cost–benefit analyses to guide sustainable policy decisions.

## Conclusion

This study found that community nurses acknowledged the mentoring model’s positive impacts, particularly in expanding practice scope, enhancing professional knowledge, and improving confidence. However, their willingness to participate was limited, primarily due to excessive workload and insufficient staffing, with dissatisfaction over performance-based pay and stress as secondary factors. Nurses also expressed strong expectations for expanded project scope, diversified support formats, clearer role boundaries, and greater patient outreach. Taken together, these findings suggest that immediate policy attention should prioritize reducing workload and optimizing human resource allocation, while subsequent reforms should refine incentive mechanisms and expand patient engagement strategies. Future research should further examine how to expand assistance projects and diversify support formats. At the policy level, immediate attention is needed to optimize workforce allocation and reduce workload, refine performance-based incentive mechanisms, and integrate nursing consortium services into medical insurance schemes. These measures would not only improve the motivation and engagement of community nurses but also strengthen the equity, accessibility, and sustainability of primary care services.

### Limitations

This study has several limitations. All participants were female directors of community nursing departments, as no male nurses in comparable roles were available and many frontline or early-career staff declined participation due to workload pressures. This homogeneity may introduce sampling bias and limit the perspectives represented. Moreover, although 162 nurses were eligible, only 10 participated (6% response rate), raising concerns about selection bias. Finally, the study was conducted in a single urban consortium in Shanghai, which may limit generalizability to rural or under-resourced regions. Nevertheless, the themes identified may offer transferable insights if adapted to local conditions. Future research should recruit more diverse participants across multiple sites and explore reasons for non-participation to strengthen the validity and applicability of findings.

## Data Availability

The original contributions presented in the study are included in the article/supplementary material, further inquiries can be directed to the corresponding authors.
